# How to Get Them off?—Assessment of Innovative Techniques for Generation and Detachment of Mature Osteoclasts for Biomaterial Resorption Studies

**DOI:** 10.3390/ijms22031329

**Published:** 2021-01-29

**Authors:** Christiane Heinemann, Josephine Adam, Benjamin Kruppke, Vera Hintze, Hans-Peter Wiesmann, Thomas Hanke

**Affiliations:** Max Bergmann Center of Biomaterials and Institute of Materials Science, Technische Universität Dresden, Budapester Str. 27, D-01069 Dresden, Germany; Josephine.Adam@tu-dresden.de (J.A.); Benjamin.Kruppke@tu-dresden.de (B.K.); Vera.Hintze@tu-dresden.de (V.H.); Hans-Peter.Wiesmann@tu-dresden.de (H.-P.W.); Thomas.Hanke@tu-dresden.de (T.H.)

**Keywords:** human monocytes, dentin discs, detachment, UpCell™, Accutase, cell scraper, thermoresponsive surface

## Abstract

The fusion process of mononuclear monocytes into multinuclear osteoclasts in vitro is an essential process for the study of osteoclastic resorption of biomaterials. Thereby biomaterials offer many influencing factors such as sample shape, material composition, and surface topography, which can have a decisive influence on the fusion and thus the entire investigation. For the specific investigation of resorption, it can therefore be advantageous to skip the fusion on samples and use mature, predifferentiated osteoclasts directly. However, most conventional detachment methods (cell scraper, accutase), lead to a poor survival rate of osteoclasts or to a loss of function of the cells after their reseeding. In the present study different conventional and novel methods of detachment in combination with different culture surfaces were investigated to obtain optimal osteoclast differentiation, yield, and vitality rates without loss of function. The innovative method—using thermoresponsive surfaces for cultivation and detachment—was found to be best suited. This is in particular due to its ability to maintain osteoclast activity, as proven by TRAP 5b-, CTSK-activity and resorption pits on dentin discs and decellularized osteoblast-derived matrix plates. In conclusion, it is shown, that osteoclasts can be predifferentiated on cell culture dishes and transferred to a reference biomaterial under preservation of osteoclastic resorption activity, providing biomaterial researchers with a novel tool for material characterization.

## 1. Introduction

Resorbability by osteoclasts is an important prerequisite that must be met in the development of bone graft substitutes that are intended to participate in the natural bone remodeling process. Consequently, a number of studies have so far dealt with biomaterial resorption in vitro and its impact on the osteoclasts [1,2,3,4,5,6,7,8,9]. Since the early stages of osteoclast investigation, the approach to harvest, produce, and isolate osteoclasts has evolved steadily, and each new method has provided important insights into the respective cellular processes. At present, however, it is still difficult to specifically study the resorption activity of mature human osteoclasts without also considering the degrees of fusion and osteoclast differentiation [10]. Usually, cells of different degree of development are obtained in one culture. This happens, for example, when osteoclasts were isolated directly from the bone tissue of young hares or chickens [11,12,13], or from murine or human osteoclast precursor cells [14,15,16]. However, in vitro studies of osteoclast bone resorption activities reveal that the cultures derived directly from bone contain osteoclasts and other cell types, such as hematopoietic cells and stromal cells, which in turn express factors affecting osteoclast activity. Therefore, another technique is to use isolated monocytes from blood instead of osteoclasts derived directly from bone, which fuse to form multinucleated cells with resorption activity [17,18]. The major disadvantage of this method, primarily for resorption studies, is that the resorption activity is highly dependent on the success of the fusion and cell maturation. It would therefore be more ideal to bring monocytes into the same state by suitable precultivation. However, this requires a successful detachment of the generated preosteoclasts, which is rarely described in the current literature due to the strong adhesion of osteoclasts and other cells of the mononuclear-phagocytic system to the substrates on which the cells were cultivated [19]. Studies on the effects of different detachment methods (trypsin, Accutase^®^, EDTA, and cell scraping) on vitality, phenotype, and function of human macrophages show that it is in principle possible to subcultivate adherent cells without loss of viability [20]. However, it also turned out that the phenotypic expression of the macrophages was influenced by the different methods of detachment [21]. In contrast, there are also studies that show that osteoclasts are resistant to detachment with trypsin [22]. Therefore, it is necessary to investigate and apply less harsh but effective methods or new techniques. Hemingway et al. found that the hydrophobic Lumox^®^ dishes enable a very high number of mature polynuclear osteoclasts to be first differentiated from peripheral blood mononuclear cells (PBMC) and then detached. These osteoclasts are highly active and produce resorption pits on dentin discs. [10].

Another promising approach is the use of thermoresponsive polymers as coatings for cell culture dishes, which are supposed to allow gentle detachment of the cells simply by changing the temperature [21,23]. A commercially available thermoresponsive cell culture dish is Nunc™ UpCell™ surface. The covalently immobilized polymer poly(N-isopropylacrylamide), PNIPAAm, is used, which exhibits a temperature-dependent phase transition in aqueous solution at 32 °C that leads to changes in the hydrophobic/hydrophilic properties [24]. Xie et al. have used this approach to detach osteoclasts, without testing its advantages or disadvantages compared to other methods [25].

The present study therefore focuses on cultivation of human monocytes on different commercially available and differently treated cell culture surfaces and investigates their impact on the monocytes with regard to adhesion and osteoclast formation potential. The predifferentiated osteoclasts are then detached from the substrate, using different detachment methods. The study provides novel insights on yield, survival, cell activity, and resorption activity after reseeding with the aim of finding the most suitable combination of cell culture surface and detachment method.

## 2. Results

### 2.1. Adhesion, Morphology, and Differentiation Capability on Different Culture Dishes

The choice of the optimal detachment method is initially preceded by analyzing the optimal culture surface for the cultivation of mature osteoclasts. In addition to the standard cell culture plate Nunclon^™^ and the thermoresponsive UpCell^™^ plates, three other plates were used for the investigation, which are designed to provide a hydrophobic substrate surface (Cellstar^®^ cell-repellent, Sarstedt Suspens, and Sarstedt Lumox^®^ Suspens).

**Figure 1:**
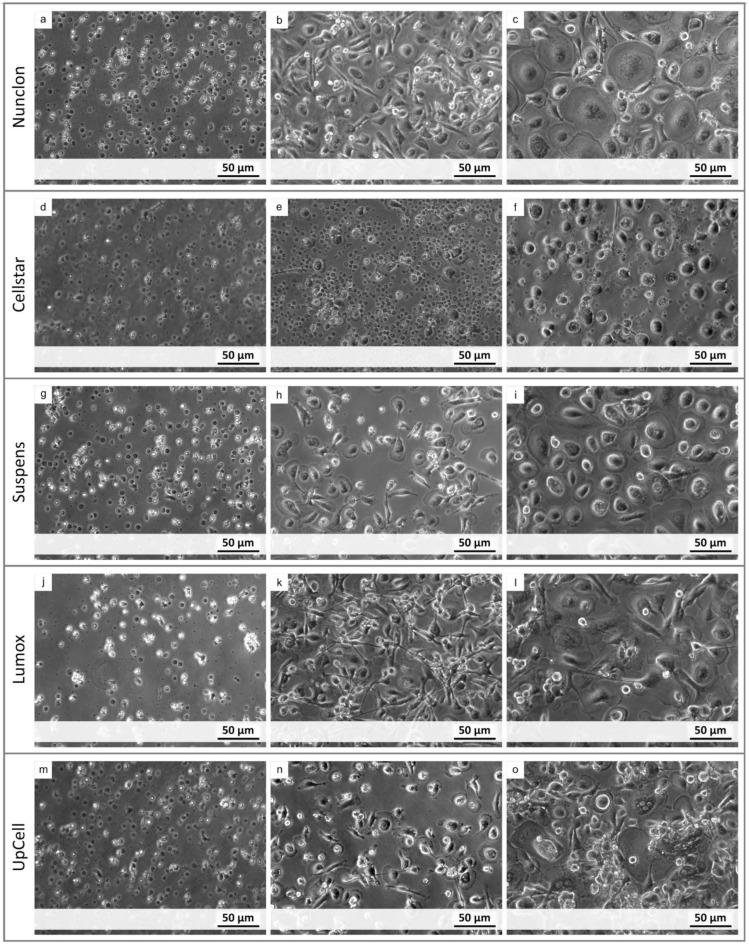
Light microscopy images of monocyte-derived osteoclasts after 1 day (left column), 4 days (middle column), and 7 days (right column) of cultivation on different cultivation surfaces (Nunclon, Cellstar, Suspens, Lumox, UpCell) (**a**–**o**).

The osteoclastic morphology was evaluated by light microscopy within the 7-day differentiation period. Differences in adhesion were marginal up to day 4 for Nunclon^™^, Suspens, and UpCell^™^. The Nunclon^™^ surface shows generally improved monocyte adhesion, which is reflected in an increased cell density at day 4 (Figure 1b). On day 7, the osteoclasts covered the entire surface. While the osteoclasts on Nunclon™ were comparatively large and round, the cells on Sarstedt Suspens appeared rather convex (Figure 1i). On UpCell™, in addition to the typical osteoclast morphology, some colony like agglomerations of cells could also be observed, which were presumably currently in the fusion process (Figure 1o). Monocytes on Lumox^®^ started with decreased adhesion on the first day after seeding, but were fully adherent up to day 4. Only a few cells were planar adherent. Most of them were elongated and showed filopodia for fusion (Figure 1k). Some multinucleated osteoclasts (up to three nuclei) could already be recognized after 4 days. After 7 days the appearance of cells on Lumox^®^ were similar to that of the Nunclon^™^ plate. Some large, round, multinucleated osteoclasts (with up to 10 cell nuclei) had formed and distributed over the entire surface of the hydrophobic culture dishes. In the Cellstar^®^ plates most monocytes remained still suspended even after 24 h owing to their cell-repellent surface. On day 4 only a small fraction of the initially seeded cells were adherent compared to the other culture dishes (Figure 1e). After 7 days, the few adherent cells had matured into osteoclasts—but with fewer than 6 nuclei—with less extensive, more convex morphology.

**Figure 2:**
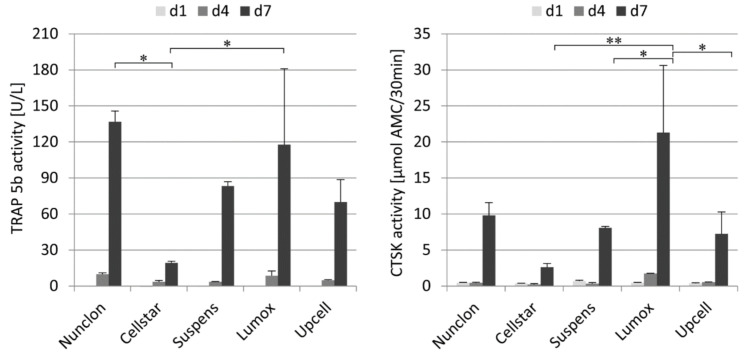
Extracellular TRAP 5b activity (left) and extracellular CTSK activity (right) of monocyte-derived osteoclasts during 7 days of cultivation on different cultivation substrates, * indicate *p* < 0.05, ** indicate *p* < 0.01.

TRAP 5b and cathepsin K (CTSK) in the medium supernatant were examined during the first 7 days of predifferentiation to evaluate the osteoclast activity of the maturation (Figure 2). On day 1 and day 4, as expected, no or very low enzyme activities of both marker proteins were measured. Then, after 7 days, both activities were significantly increased on all culture plates with culture plate specific differences. The osteoclasts on Cellstar^®^ expressed significantly lower activity values owing to the fact that only few cells adhered. Extracellular TRAP 5b and CTSK activity were significantly enhanced on day 7 by osteoclasts on Lumox^®^ culture dishes and Nunclon™. For these dishes, it is obvious that the high activity values of both markers are caused by the high cell number of adhering osteoclasts, which is confirmed by the light microscopic images.

### 2.2. Yield and Survival after Reseeding

**Figure 3:**
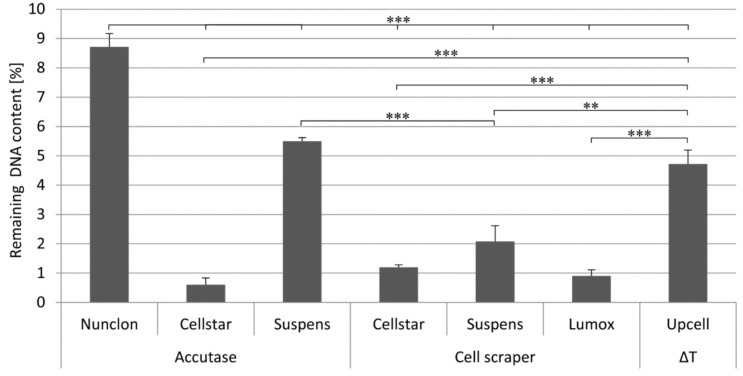
Remaining DNA content on different cultivation surfaces after different detachment procedures as determined by DNA measurement, ** indicate *p* < 0.01, and *** *p* < 0.001.

The efficiency of the detachment was indirectly determined by quantifying the remaining DNA content on the culture dishes after the detachment procedure (Figure 3). With all detachment methods making use of the cell scraper, the previously adherent cells were almost completely detached. Likewise, only a small fraction of cells on Cellstar^®^ remained after the Accutase treatment. In contrast, on Sarstedt Suspens and Nunclon™ dishes 5–9% remaining DNA was measured on the surface after the Accutase treatment. Nunclon™ shows the significantly highest value, thus it is also the plate with the strongest cell adhesion. When using the temperature-responsive UpCell™ dish, on which the cells are detached from the surface by simply rinsing with medium after the plate has been cooled down to 20 °C by adding tempered medium first, only 5% of the remaining DNA content was measured.

**Figure 4:**
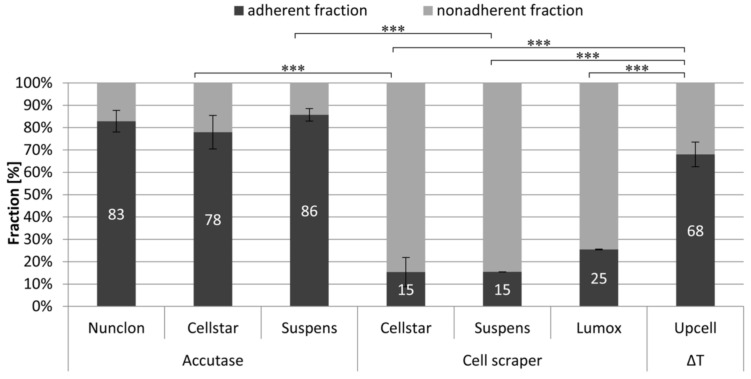
Adherent and non-adherent fraction of mature osteoclasts 1 day after reseeding on Nunclon^TM^ reference plates determined by DNA measurement in lysates (adherent fraction) and supernatants (nonadherent fraction), *** indicate *p* < 0.001.

An impression of the survival rate of the cells after detachment can be gained by the adherence after reseeding (Figure 4). The detached cells were reseeded as predifferentiated osteoclasts on Nunclon^TM^ reference plates. The measurement of survival rate as well as the amount of nonadherent cells was used for calculating the percentages of the adherent and nonadherent fractions. These showed that the already well differentiated osteoclasts of the samples UpCell™, Suspens/Accutase, and Nunclon™/Accutase strongly adhered on the first day after reseeding and were rarely suspended in the medium. However, even the very few cells that grew on the Cellstar^®^ surface were also characterized by particularly strong adherence. In contrast, the cells removed by means of the cell scraper were predominantly damaged, so that they were measurable as dead cells in the supernatant.

### 2.3. Cell Activity and Resorption after Reseeding

**Figure 5:**
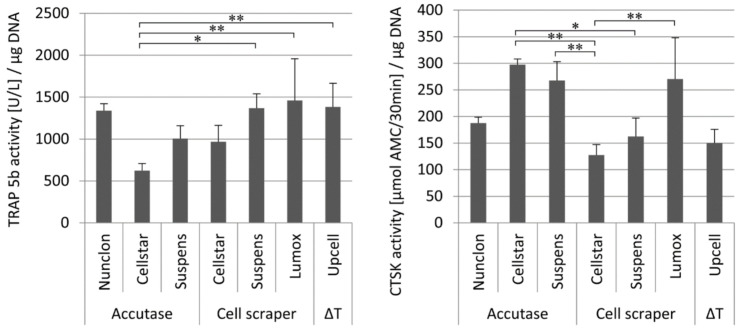
Intracellular TRAP 5b activity (**left**) and intracellular CTSK activity (**right**) of reseeded mature osteoclasts depending on the different detachment methods after 7 days of cultivation on Nunclon^TM^ reference plates, * indicate *p* < 0.05, ** indicate *p* < 0.01.

**Figure 6:**
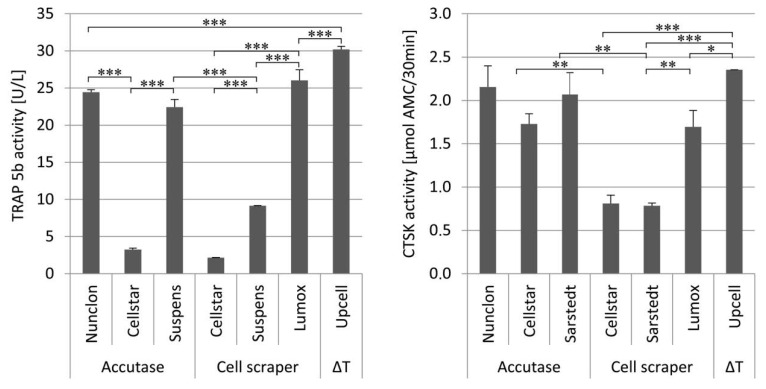
Extracellular TRAP 5b activity (**left**) and extracellular CTSK activity (**right**) of the reseeded mature osteoclasts depending on the different detachment methods after 7 days of cultivation on dentin discs, * indicate *p* < 0.05, ** indicate *p* < 0.01, and *** *p* < 0.001.

The enzyme activity of TRAP 5b and CTSK were investigated in order to evaluate the effect of the detachment procedures on the remaining cell activity after reseeding. The intracellular enzyme activity was measured solely for cells cultivated on Nunclon^TM^ reference plates (Figure 5). The extracellular enzyme activity was measured to determine the resorptive activity of osteoclasts on dentin discs (Figure 6).

Seven days after reseeding, osteoclasts detached by the different techniques and reseeded onto Nunclon^TM^ reference plates showed quite uniform TRAP 5b activity with the significant exception of Cellstar^®^/Accutase which caused reduced activity. On the contrary, CTSK activity of the three detachment methods Cellstar^®^/Accutase, Suspens/Accutase, and Lumox^®^ showed the highest activity after reseeding, respectively.

The measurement of the TRAP 5b activity in the supernatant of the osteoclast culture on dentin discs demonstrated that UpCell™ showed significantly increased values in this case compared to the detachment methods Nunclon^TM^/Accutase, Suspens/Accutase, and Lumox^®^. The measured CTSK activities expressed by osteoclasts into supernatant during cultivation on dentin discs showed generally a high level. Once again UpCell™ showed the best results. As had already been seen for TRAP 5b activity, Cellstar^®^/cell scraper and Suspens/cell scraper showed also significantly reduced CTSK activities compared to the other detachment methods.

**Figure 7:**
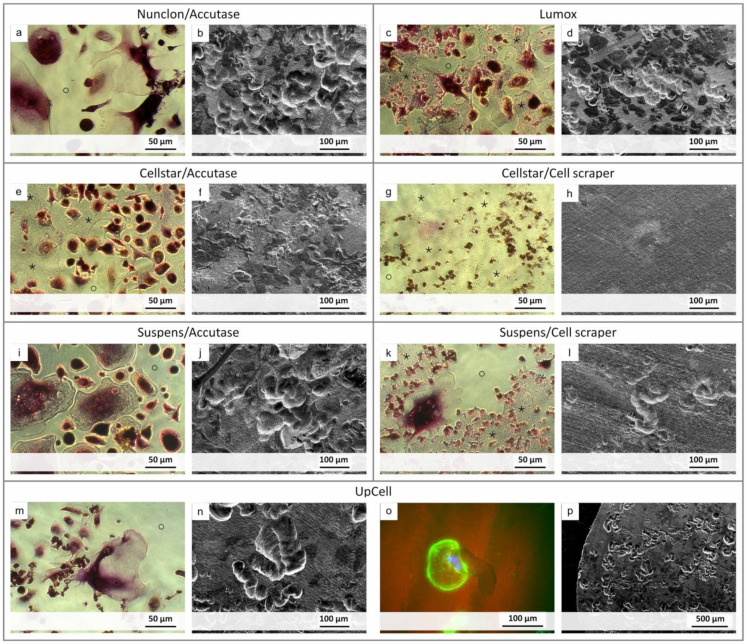
Images showing the resorption activity of the detached osteoclasts after reseeding. Light micrographs after TRAP staining of the cells show resorption (marked as o) of the decellularized osteoblast-derived matrix layer (marked as *) ((**a**,**c**,**e**,**g**,**I**,**k**,**m**) for each detachment method). SEM images show resorption lacunas on dentin discs ((**b**,**d**,**f**,**h**,**j**,**l**,**n**,**p**) respectively). Special focus was put on UpCell-generated osteoclasts, where one is exemplary shown in a resorption lacuna (fluorescence image with actin skeleton shown in green and the nuclei in blue, UpCell (**o**)).

To demonstrate the resorption activity, the premature osteoclasts were reseeded and cultured on decellularized osteoblast-derived matrix plates and also on dentin discs (Figure 7). The cells on the matrix plates were additionally TRAP 5b stained for visualization of osteoclast activity. Nonetheless, the morphology of the cells alone indicates clear differences between the individual methods. In addition, it could be seen which detachment method allowed reseeding of mature and active osteoclasts capable of resorbing the matrix. With Nunclon™/Accutase, Suspens/Accutase, and UpCell™, the matrix was no longer detectable after 7 days, since it had been resorbed by the osteoclasts. Furthermore, the former two show the largest osteoclasts, followed by UpCell™ and Lumox^®^, for which also significantly smaller osteoclasts were found. Those were attributed to subsequent fusion after the reseeding process. Precisely these two methods, UpCell™ and Lumox^®^, showed colony-like agglomerates of cells that had not completely fused on the day of detachment.

In contrast, the Cellstar^®^/cell scraper method displayed predominantly mononuclear cells with weak TRAP staining. The matrix layer showed no defects even after 7 days. The cells seemed to lie on the matrix instead of resorbing it. This was confirmed by the study on dentin disc, where no resorption lacunae were found either.

Many mononuclear cells were also found for the method using Suspens/cell scraper, although very large osteoclasts could be generated on the Suspens plates during first seeding. Obviously, the negative impact of the cell scraper was considerable, so that only a few large osteoclasts were present, which are responsible for the resorption of the matrix. This is also confirmed by the few resorption lacunae on the dentin discs. Likewise, with Cellstar^®^/Accutase, a large part of the decellularized matrix remained intact after 7 days culture time of reseeded osteoclasts. Here, the osteoclasts showed comparatively small morphology owing to the hydrophobic cell-repellent surface of Cellstar^®^ during first cultivation. Despite osteoclast-like morphology with existing (albeit low) TRAP 5b activity, no resorption lacunae were found on the dentin discs. Only the remnants of the cells were visible on the surface. In summary, the most prominent resorption lacunae on dentin discs were caused by cells harvested by the following methods: Nunclon™/Accutase, Suspens/Accutase, and UpCell™. For the latter, a larger section of the dentin discs is shown as an example, proving the large number of equally distributed resorption lacunae on the entire dentin disc surface (Figure 7p)).

The cells on the dentin discs were further prepared for examination by fluorescence microscopy for simultaneous imaging of osteoclasts and resorption lacunae. As exemplarily shown, the osteoclast generated with the UpCell™ method is located at the end of its resorption lacunae (Figure 7o).

## 3. Discussion

One of the major disadvantages of in vitro resorption studies of biomaterials is the necessary formation of active resorbing osteoclasts from a larger number of monocytes, which can be strongly restricted by the morphology of the different specimens and the topography of the surface. Thus, it may be advantageous to skip the fusion process and use mature, differentiated osteoclasts for in vitro resorption studies. It should be the method of choice to precultivate monocytes on cell culture dishes, remove them after differentiation, and reseed them on various biomaterials for bone regeneration. This would represent a uniform, defined initial state for material and resorption analysis. The aim of this study was to optimize the most favorable combination of culture plate cultivation and detachment method to achieve the best possible yield and cell activity after reseeding.

The detachment of mature osteoclasts is difficult owing to their strong adherence to standard tissue culture plates and their resistance to trypsin [22]. Detachment is often accompanied by significant decrease in cell number. In accordance with the fact that hydrophobic culture dishes are known for better detachment of osteoclasts [10], three different culture dish types were analyzed, which provide a hydrophobic surface according to the manufacturers specifications. These are the cell-repellent culture dishes from “Cellstar^®^ cell-repellent” as well as “Sarstedt Suspens” and “Sarstedt Lumox^®^ Suspens”. Those are intended to prevent growth of cells and were actually specially developed for cultivation of nonadherent cells in suspension. In addition, the standard cell culture dish Nunclon™ with optimized properties for adherent cells was selected as reference. Furthermore, a novel, thermoresponsive cell culture surface (UpCell™) was examined with regard to the hypothesis that a particularly gentle detachment is possible. The latter offers good properties with regard to cell adhesion at incubator temperature and promises simple detachment of the cells after cooling to 20 °C.

Initially, the focus of this study was put on the ability of the monocytes to adhere to the respective dish surfaces giving optimal conditions for formation and differentiation of mature osteoclasts. This is crucial for the effectiveness of the combination of plate and detachment method. On day 1 after cell seeding, microscopic observation showed that all cells had adhered sufficiently, except those that had been seeded on the cell-repellent Cellstar^®^ dishes. The surface of the Cellstar^®^ dishes did not permit adhesion of the monocytes, thus only a small part was able to adhere at all. Without the possibility to adhere the monocytes on the Cellstar^®^ dishes hardly fused and differentiated until the end of the first week of precultivation. Despite visual proof of osteoclastic morphology, only few adherent cells could mature into osteoclasts with a maximum of three nuclei. During analysis of the resorption lacunae, cells originating from Cellstar^®^ dishes, which were only marginally differentiated prior to detachment, proved to be the least resorbing cells after reseeding on decellularized osteoblast-derived matrix plates as well as dentin discs. Neither the detachment with cell scrapers nor the detachment with Accutase^®^ led to osteoclasts originating from Cellstar^®^ able to resorb the dentin discs. Therefore, the use of Cellstar^®^ for osteoclast generation and resorption studies is not advisable. In contrast to these results, Klinder et al. recommended the use of Cellstar^®^ surfaces for enriching monocytes from PBMCs for improved adhesion and differentiation into macrophages. Here they presented Cellstar^®^ as the metfhod of choice in combination with RPMI medium. However, this does not concern the differentiation to osteoclasts and their resorption capacity, which were not subject of their study [26].

In contrast to osteoclasts from Cellstar^®^, the cells on all other dishes showed very good progress in their osteoclastic fusion and differentiation, as determined by light microscopy and TRAP 5b activity. In general, Nunclon™ plates represent a reference of optimal growth conditions for adherent cell cultures, so that successful differentiation was to be expected. In the case of the slightly hydrophobic cultivation surfaces of Lumox^®^ culture dishes, the high TRAP 5b activity indicates that their gas-permeable soil offers comparatively good growth conditions. It has been shown earlier by Gretzer et al. that monocytes are able to bind to surfaces with higher hydrophobicity than Nunclon™ [27]. Hemingway et al. also used Lumox^®^ to cultivate osteoclasts and have shown positive results in differentiation and activity [10]. In our study, however, the osteoclasts on Lumox did not detach by simply tapping the culture plate as described by Hemingway et al., but had to be removed by using the cell scraper. Bernhardt et al. reported similar experiences with Lumox^®^ [28]. Despite the use of cell scrapers, the osteoclasts detached from Lumox^®^ culture plates showed comparably good resorption activity. The other methods Sarstedt Suspens/cell scrapers and Cellstar^®^/cell scrapers resulted in only a few remaining cells on the surface. Nevertheless, this very invasive procedure had a negative effect on the survival rate, vitality, and resorption activity of the osteoclasts after reseeding. Osteoclasts detached by cell scraper showed the least ability to resorb dentin.

Accutase-mediated detachment methods showed very good results in terms of yield and survival, despite the necessary centrifugation step. The detachment with Accutase is also the method of choice for many researchers [29,30], but certainly also because there are only few studies on optimized detachment methods. In terms of cell activity and resorption capacity, the variants Nunclon™/Accutase and Suspens/Accutase as well as Lumox^®^ and UpCell™ showed the best results.

The optimal combination of cultivation dish and detachment technique was determined under different aspects in order to achieve a high amount of resorbing mature osteoclasts. From our point of view, the cultivation and detachment using UpCell™ dishes offers a major advantage over all other methods. On the one hand, the enzymatic influence of Accutase with the associated centrifugation step and the resulting cell stress is eliminated. On the other hand, the different multiwell sizes of the UpCell™ products offer the possibility to develop a defined amount of monocytes per well into mature osteoclasts and to remove this amount in a defined way and use it for further investigations.

Regarding avoidance of stress by Accutase and centrifugation, Rennert et al. [31] also came to this conclusion, when investigating the influence of different thermoresponsive culture surfaces on macrophages [31]. Furthermore, studies on macrophages also indicated that enzymatic detachment techniques, although shown to be beneficial in terms of cell yield and viability, can induce profound phenotypic and functional changes [23].

Regarding the second advantage of having a defined cell quantity in different well sizes, it can be concluded that an additional counting step is not necessary. This is even more important, as a quantification with a hemocytometer is rather difficult owing to the different size of the resuspended osteoclasts, because polynuclear cells remain in suspension in the middle large square and can no longer be distributed evenly over the entire Neubauer chamber. In addition, cultivation in multiwell plates offers the possibility to transfer the mature differentiated osteoclasts from one well after cooling to 20 °C by resuspension and reseeding on one sample each. Thus, the actual nuclei number per sample can be determined quantitatively and allows cell count-based quantification of enzyme activities as well as the resorption lacunae.

## 4. Materials and Methods

### 4.1. Cultivation of Monocytes to Mature Osteoclasts on Different Culture Dishes

Human monocytes were isolated from human buffy coats as described here. The buffy coats were diluted 1:1 with PBS containing 2 mM EDTA and 0.5% BSA and coated on leucosep tubes with Ficoll-Paque PREMIUM 1.073 g/L (GE Healthcare, München, Germany). After a centrifugation step at 836 rcf for 20 min, PBMC fraction was collected and additionally separated with a density gradient of 1.063 g/mL (prepared by dilution of Ficoll-Paque 1.077 g/L (GE Healthcare) with PBS EDTA/BSA) for 15 min at 350 rcf to remove the platelets. Mononuclear cells were washed with PBS/EDTA/BSA and centrifuged for 8 min at 300 rcf. Finally, monocytes were purified by magnetically activated cell sorting via negative selection with a monocyte isolation kit II according to the manufacturer’s instructions (Miltenyi, Bergisch Gladbach, Germany).

Monocytes were seeded with a density of 2 × 10^6^ per 35 mm Ø dish on the different culture dishes. This is referred to as first seeding. Osteoclastic differentiation was induced by the addition of 50 ng/mL M-CSF and 50 ng/mL RANKL to the medium. The medium was changed on day 4. Medium and all supplements were obtained from Biochrom, Berlin, Germany. The following cell culture dishes were investigated (Table 1):

***Nunclon™ Delta:*** The Nunclon^™^ culture dish (Thermo Scientific, Bremen, Germany), modified to optimize cell adhesion, has a fully synthetic, specially treated surface that makes the otherwise strongly hydrophobic polystyrene more hydrophilic. Thus, cell adhesion and cell growth of a wide variety of cell types is promoted and facilitated.

***CELLSTAR^®^ Cell-repellent surface****:* The cell-repellent CELLSTAR well-plates (Greiner Bio-One, Frickenhausen, Germany) have a chemically modified surface, developed in particular to effectively prevent the attachment of semiadherent and adherent cell lines. The highly hydrophobic surface is therefore particularly suitable for the cultivation of e.g., macrophages.

***Tissue culture Suspension:*** Suspension culture dishes (Sarstedt, Nümbrecht, Germany) were developed for the cultivation of nonadherent cells. Their hydrophobic surface should prevent cell losses, which could occur during cell harvest for planned subcultivation if undesired microadhesions of the cells occur.

***Lumox^®^ Suspens:*** The Lumox^®^ cultivation dishes (Sarstedt, Nümbrecht, Germany) have a hydrophilic, gas-permeable, 25 µm thick film base, which is intended to guarantee optimum gas exchange through extremely short diffusion paths. The gas exchange, which is not possible through the bottom of conventional cell culture dishes, is intended to enable cell growth even of cells that are difficult to cultivate, as well as to generally optimize the growth conditions of different adherent cell types.

***UpCell™ surface*:** The temperature-sensitive surfaces of the UpCell™ culture dishes (Thermo Scientific, Bremen, Germany) were specially developed for the nonenzymatic harvesting of adherent cells. In order to maintain the viability of the cells and surface proteins to be dissolved during harvest, the culture must be brought to room temperature. The immobilized polymer poly(N-isopropyl acrylamide) (PIPAAm), which forms a uniform, thin layer on the bottom of the UpCell™ culture vessel, is slightly hydrophobic at 37 °C, allowing cells to accumulate and grow. If the temperature of the culture is reduced to below 32 °C, the PIPAAm layer becomes strongly hydrophilic, binds water, swells, and releases the adherent cells again.

### 4.2. Detachment of Mature Osteoclasts Using Various Techniques

On day 7 (d7) after first seeding and osteoclast induction, the cells were detached from their respective cultivation surfaces using the following procedures.

***Temperature-induced detachment:*** This method of detachment, used exclusively on UpCell™ cultivating plates, involved the removal of the old medium and rinsing with warm (37 °C) PBS. According to the manufacturer, after the addition of medium tempered to 20 °C and a resting period of 20 min, the cells detach from the surface.

***Detachment with Accutase^®^****:* Accutase^®^ (Biochrom, Berlin, Germany) was added at 37 °C for 30 min after the old medium had been removed and the cells were rinsed with warm PBS. The resulting enzyme cell suspension was finally centrifuged at 300 rcf for 3 min and the remaining cell pellet was resuspended in fresh medium.

***Detachment by scraping:*** The old medium was removed, the dishes rinsed with 37 °C warm PBS and fresh medium added before the cells were carefully removed from the surface with a cell scraper.

### 4.3. Analysis of Yield, Survival, Cell Activity, and Resorption after Reseeding

The cells were resuspended in fresh medium and a second seeding (reseeding) was performed on Nunclon™ (Delta) reference plates (48 well) with reduced amounts of M-CSF (25 µg/mL) and RANKL (25 µg/mL). To investigate resorption, the precultured osteoclasts were further cultivated on dentin discs (Immunodiagnostic Systems GmbH, Frankfurt, Germany) and decellularized osteoblast-derived extracellular matrix plates (SAOS-mineralized ECM plates) [32].

#### 4.3.1. Biochemical Analyses

In all experiments, at specific time points medium supernatants were collected and the plates with the adherent cells were washed with PBS and frozen at −80 °C until biochemical analysis. The latter were then lysed with 1% Triton X-100 (Sigma, Steinheim, Germany) in PBS on the day of analysis. A SpectraFluor Plus microplate reader (Tecan, Crailsheim, Germany) was used for all colorimetric measurements.

***DNA-assay:*** The DNA amount was analyzed with the Quant-iT™ PicoGreen^®^ dsDNA reagent according to the manufacturer’s instructions. Fluorescence intensity was measured at 485/535 nm excitation/emission wavelengths and correlated with a DNA calibration curve.

***TRAP-activity-assay:*** The activity of tartrate-resistant acid phosphatase 5b (TRAP 5b) was measured according to a slightly modified protocol by Janckila et al. using naphthol ASBI phosphate (N-ASBI-P, Sigma) as substrate [33]. Cell lysate and TRAP 5b reaction buffer (2.5 mM N-ASBI-P, 50 mM Na-tartrate (Sigma), 2% NP-40 (Sigma), 1% ethylene glycol monomethyl ether (Sigma) in 0.1 M Na-acetate (Sigma) buffer, pH 6) were incubated in microtiter plates at 37 °C. After 1 h the enzymatic reaction was stopped by the addition of 0.1 M NaOH and the fluorescence was measured at 405/535 nm. The fluorescent units were converted into activities using a TRAP 5b standard.

***Cathepsin K-assay:*** The activity of cathepsin K was quantified using the substrate Z-LR-AMC (Enzo Life Sciences, Lörrach, Germany) dissolved in DMSO. For the measurements, the cell lysates or supernatants were mixed with 100 µM of the substrate dissolved in substrate buffer (0.1 M sodium acetate buffer containing 4 mM EDTA and 4 mM DTT, pH 5.5) and incubated in microtiter plates at 37 °C for 30 min. The reaction was stopped by adding 0.1 M iodoacetic acid in 0.1 M TRIS buffer and the fluorescence was measured at 365/440 nm. The calibration was performed with a dilution series of 7-amino-4mathylcoumarin (AMC) in DMSO.

#### 4.3.2. Microscopic Analyses

***Light microscopy:*** Light microscopy imaging of the cells during the culture period was performed using a Zeiss Axiovert 25 (Zeiss, Jena, Germany) equipped with a digital camera (Canon, Tokyo, Japan). For additional TRAP staining, the cells were fixed with 3.7% formaldehyde after 7 days of reseeding. The Acid Phosphatase, Leukocyte (TRAP) Kit (Sigma) was used for visualization according the manufacturer’s instructions.

***Scanning electron microscopy:*** Scanning electron microscopy (SEM) was performed with the dentin discs to visualize resorption lacunae. The samples were mounted on aluminum stubs and coated with carbon in a Balzers SCD 050 coater. A Philips ESEM XL 30 SEM operating in HiVac mode at 3 kV was used for imaging by secondary electron detection.

***Fluorescence microscopy:*** To visualize the actin cytoskeleton and the nuclei of the osteoclasts on the dentin discs, the cells were fixed with 3.7% formaldehyde, permeabilized with 0.2% Triton-X-100 in PBS and stained with Alexa-Fluor 488 phalloidine and DAPI. Fluorescence microscopy was performed using the Axioskop 2FSmot fluorescence microscope (Zeiss, Jena), equipped with an Axioscan camera (Zeiss) and controlled by the Axio Vision 3.1 (Zeiss) software.

### 4.4. Statistics

All measurements were collected at least in triplicates and expressed as mean ± standard deviations. One-way ANOVA with Tukey post hoc analysis was employed for comparison of selected samples to assess significant differences with *p* values less than 0.05 considered significant and indicated by one asterisk, while ** indicate *p* < 0.01, and *** *p* < 0.001.

## 5. Conclusions

Precultivation of mature osteoclasts in multiwell dishes in order to detach them without damage and further cultivate them on biomaterials offers a new possibility for in vitro resorption studies independent of the fusion process. In the present study, different culture dishes and detachment methods were combined to identify the best in terms of yield, survival rate, osteoclast enzyme activity, and resorption capacity.

Two of them turn out to be very effective: a conventional variant—Accutase which is often used owing to a lack of alternatives—and an innovative method—the usage of UpCell™ plates. The latter, uninfluenced by enzymes, centrifugation, or mechanical scraping, offers new possibilities, especially for subsequent resorption studies with regard to better quantification through the use of multiwell dishes. This innovative method allows the investigation of the influence of the scaffold architecture and the material composition specifically on the resorption activity after fusion. In this context, not only the improved resorption performance of the prematured osteoclasts is an advantage, but also a better quantification of the results. The more uniform osteoclast population is much better suited for standardized analyses.

## Figures and Tables

**Table 1:** Manufacturer-specific catalog numbers of the used culture dishes.

Name	Description	Manufacturer/Supplier	Catalog No.
Nunclon^™^	Nunclon^™^ Delta	Nunc^™^/Thermo Scientific^™^	150318
Cellstar^®^	CELLSTAR^®^ Cell-repellent	Greiner Bio-One	627979
Suspens	Tissue Culture Suspension	Sarstedt	83.3900.500
Lumox^®^	Lumox^®^ Suspension	Sarstedt	94.6077.333
UpCell^™^	UpCell™ Surface	Nunc^™^/Thermo Scientific^™^	174904

## Data Availability

All data generated or analysed during this study are included in this published article.
